# Modeling cellular responses to serum and vitamin D in microgravity using a human kidney microphysiological system

**DOI:** 10.1038/s41526-024-00415-2

**Published:** 2024-07-09

**Authors:** Kevin A. Lidberg, Kendan Jones-Isaac, Jade Yang, Jacelyn Bain, Lu Wang, James W. MacDonald, Theo K. Bammler, Justina Calamia, Kenneth E. Thummel, Catherine K. Yeung, Stefanie Countryman, Paul Koenig, Jonathan Himmelfarb, Edward J. Kelly

**Affiliations:** 1 Department of Pharmaceutics, University of Washington, Seattle, WA USA; 2 Department of Environmental and Occupational Health Sciences, University of Washington, Seattle, WA USA; 3 Department of Pharmacy, University of Washington, Seattle, WA USA; 4 Kidney Research Institute, Seattle, WA USA; 5 BioServe Space Technologies, University of Colorado, Boulder, CO USA; 6 Present Address: RayzeBio, San Diego, CA USA

**Keywords:** Acute kidney injury, Cell biology

## Abstract

The microgravity environment aboard the International Space Station (ISS) provides a unique stressor that can help understand underlying cellular and molecular drivers of pathological changes observed in astronauts with the ultimate goals of developing strategies to enable long- term spaceflight and better treatment of diseases on Earth. We used this unique environment to evaluate the effects of microgravity on kidney proximal tubule epithelial cell (PTEC) response to serum exposure and vitamin D biotransformation capacity. To test if microgravity alters the pathologic response of the proximal tubule to serum exposure, we treated PTECs cultured in a microphysiological system (PT-MPS) with human serum and measured biomarkers of toxicity and inflammation (KIM-1 and IL-6) and conducted global transcriptomics via RNAseq on cells undergoing flight (microgravity) and respective controls (ground). Given the profound bone loss observed in microgravity and PTECs produce the active form of vitamin D, we treated 3D cultured PTECs with 25(OH)D_3_ (vitamin D) and monitored vitamin D metabolite formation, conducted global transcriptomics via RNAseq, and evaluated transcript expression of CYP27B1, CYP24A1, or CYP3A5 in PTECs undergoing flight (microgravity) and respective ground controls. We demonstrated that microgravity neither altered PTEC metabolism of vitamin D nor did it induce a unique response of PTECs to human serum, suggesting that these fundamental biochemical pathways in the kidney proximal tubule are not significantly altered by short-term exposure to microgravity. Given the prospect of extended spaceflight, more study is needed to determine if these responses are consistent with extended (>6 months) exposure to microgravity.

## Introduction

The International Space Station (ISS) is a modular spacecraft replete with stressors that challenge the bounds of human physiology. Astronauts aboard the ISS live in a tight-quarter, enclosed, near-weightless environment in low Earth orbit. Astronauts face superterrestrial levels of ionizing radiation, disruption of circadian rhythms, and encephalic fluid redistribution^1,2^. Because of microgravity-induced physiological changes, astronauts commonly exhibit muscle atrophy, ophthalmic disorders, serum chemistry alterations, and bone demineralization^3–6^. Many of these physiological changes mirror disease states on Earth, including age-related changes in telomere maintenance and hormonal perturbations^7,8^. As such, the microgravity environment has been proposed as a unique stressor that can help understand underlying cellular and molecular drivers of pathological changes observed in astronauts with the ultimate goal of developing strategies to enable long-term spaceflight and better treatment of diseases on Earth^9^. We used the unique environment of the ISS to evaluate the effects of microgravity on the kidney response to serum exposure and biotransformation of vitamin D.

The kidneys play an essential homeostatic function by filtering out waste products of cell metabolism. While small waste molecules are freely filtered, larger serum proteins such as albumin and immunoglobulins are efficiently retained within the circulation. However, the selectivity of the kidney filtration barrier is disrupted in several common diseases, resulting in the spillage of serum proteins into the urine (proteinuria). It is estimated that 10% of adults in the United States have elevated levels of serum-derived albumin detectable in their urine^10^. Data regarding the renal handling of filtered protein (such as albumin) in astronauts in flight is conflicting; while it was initially reported that urinary excretion of albumin was increased in astronauts in flight, follow-up studies showed decreased urinary excretion of albumin^11–13^.

Whether proteinuria can directly activate injury in kidney tubules or exacerbate disease progression is controversial^14^. Ground-based studies have shown that serum, but not its major protein component albumin, induced tubular injury and secretion of pro-inflammatory cytokines and matrix modifying enzymes, demonstrating a causal role for serum proteins in tubular injury^15^. To test whether the additional stressor of microgravity alters the pathologic response of the proximal tubule to serum exposure, we treated human proximal tubule epithelial cells (PTECs) cultured in a microphysiological device with human serum and measured biomarkers of toxicity and inflammation (KIM-1 and IL-6) and conducted global transcriptomics via RNAseq on cells undergoing flight (microgravity) and respective controls (ground).

The kidney may play an important role in bone loss in microgravity through altered metabolism of 25-hydroxy vitamin D_3_ (25(OH)D_3_) to its most biologically active form, 1α,25-dihydroxy vitamin D_3_ (1α,25(OH)_2_D_3_) or inactive degradation products such as 24R,25 dihydroxy vitamin D_3_ (24R,25(OH)_2_D_3_. 1α,25(OH)D_3_ is important for bone homeostasis, primarily through regulation of uptake of calcium in the intestine and modulation of osteoclast number and activity^16^. Despite dietary supplementation of vitamin D_3_ and plasma levels of 25(OH)D_3_ remaining constant, plasma levels of 1α,25(OH)_2_D_3_ in astronauts in flight decrease over time^17,18^. At the same time, absorption of calcium in the intestine is impaired^17^. The kidney is the primary site for bioactivation of 25(OH)D_3_ to 1α,25(OH)_2_D_3_, via cytochrome P450 27B1 (CYP27B1).

The kidney can also metabolize 25(OH)D3 and 1α,25(OH)_2_D_3_ to inactive products via cytochromes P450 24A1 (CYP24A1) via CYP3A5^16^. In addition, the kidney maintains the levels of 1α,25(OH)2D3 through an autocrine mechanism, whereby 1α,25(OH)_2_D_3_ activates the vitamin D receptor (VDR) leading to the induction of *CYP24A1*. Thus, microgravity could decrease plasma levels of 1α,25(OH)_2_D_3_ by (1) decreasing renal CYP27B1 activity, (2) increasing renal CYP24A1 activity, or (3) increasing renal CYP3A5 activity. To test whether microgravity affects the transcript expression or activity of CYP27B1, CYP24A1, or CYP3A5, we treated proximal tubule epithelial cells (PTECs) cultured in a microphysiological device with 25(OH)D_3_ and monitored metabolite formation and conducted global transcriptomics via RNAseq on cells undergoing flight (microgravity) and their control (ground).

## Results

### PT-MPS platform and perfusion system

**Fig. 1:**
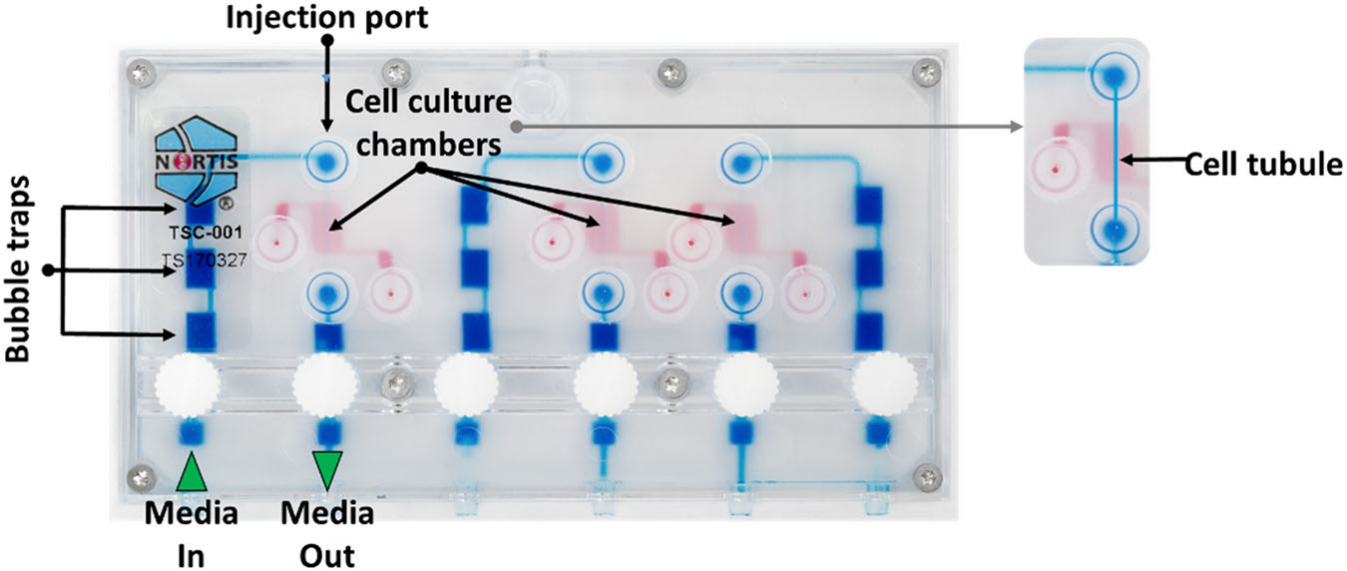
Nortis Triplex chip showing flow path, bubble traps, injection ports, and three individually perfused cell culture chambers.

Nortis microfluidic chips are molded from polydimethylsiloxane, a semi-transparent, flexible, generally bio-compatible, and gas-permeable silicone polymer (Fig. 1). While the footprint of the Nortis Triplex chip is relatively small, the equipment required for perfusion including chip platform, shelves, docking station, and pneumatic pump are relatively large. In order to reduce the footprint of the Triplex chip during perfusion and meet the levels of containment required by the National Aeronautics and Space Administration (NASA), we partnered with BioServe Space Technologies to design, machine, and fabricate a novel perfusion platform as previously described^19^.

### Experimental design and loss of devices to mold contamination

**Fig. 2:**
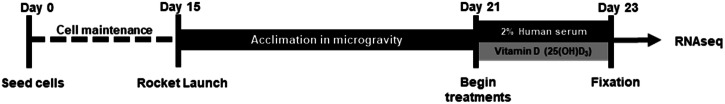
General experimental scheme. After 6 days of acclimation in microgravity, the maintenance media cassettes were switched to treatment media cassettes containing either maintenance media, 2% human serum, or 3 µM vitamin D binding protein (DBP) + 1.5 µM vitamin D (25(OH)D_3_) and treated for 2 days before fixation with either RNAlater for RNAseq or formalin for immunocytochemistry.

After delivery and installation on board the ISS, cells were acclimated to microgravity for 6 days (Fig. 2). Maintenance media cassettes were switched to treatment media cassettes containing either maintenance media, 2% human serum, or 3 µM vitamin D binding protein (DBP) + 1.5 µM vitamin D (25(OH)D_3_) and treated for 48 h prior to fixation with either RNA later for RNAseq or formalin. Ground controls were conducted using minute-to-minute time matching with on-station timing, with a 36 h delay. During the disassembly of the chips from the housing unit, mold was observed on the exterior of several chips near the matrix port, cell seeding port, and edges. Mold was also observed within the flow path of some devices.

Media overflow from the injection port was noted from 9.7% (14/144) of the channels before integration into the BioServe perfusion platform and may have contributed to the contamination. Consequently, channels that had visible mold, issues with RLT lysis buffer perfusion during RNA isolation (due to potential blockage by mold), or notably discolored effluents were excluded from the analyses. In total, 65.3% (47/72) and 66.7% (48/72) of the ground and flight samples were included in effluent analyses, respectively. 72.2% (39/54) and 61.1% (33/54) of the ground and flight samples were analyzed by RNAseq, respectively. 94.4% (17/18) and 55.6% (10/18) of the ground and flight samples were used for the analysis of vitamin D metabolites, respectively. The number of usable samples for each donor separated by treatment and condition (ground vs. flight) is summarized in Table 1.

**Table 1:** Number of samples usable for effluent analysis and RNAseq analysis

		Effluent analysis samples				RNAseq analysis samples			
		Donors				Donors			
		M1	M2	F1	F2	M1	M2	F1	F2
Ground	Media	2/9	0/9	5/12	6/6	2/6	0/3	3/6	3/
	Vitamin D	5/6	3/3	6/6	3/3	5/6	3/3	6/6	2/3
	2% Human serum	5/6	3/3	6/6	33	5/6	2/3	6/6	2/3
Flight	Media	6/9	7/9	10/12	0/6	3/6	3/3	4/6	0/3
	Vitamin D	5/6	0/3	3/6	2/3	4/6	0/3	2/6	2/3
	2% Human serum	5/6	2/3	6/6	2/3	6/6	2/3	5/6	2/3

Fractions represent the number of samples included in each analysis over the total number of samples at the beginning of the experiment.

### Transcriptional response of PTECs to 2% human serum in ground and flight conditions

**Fig. 3:**
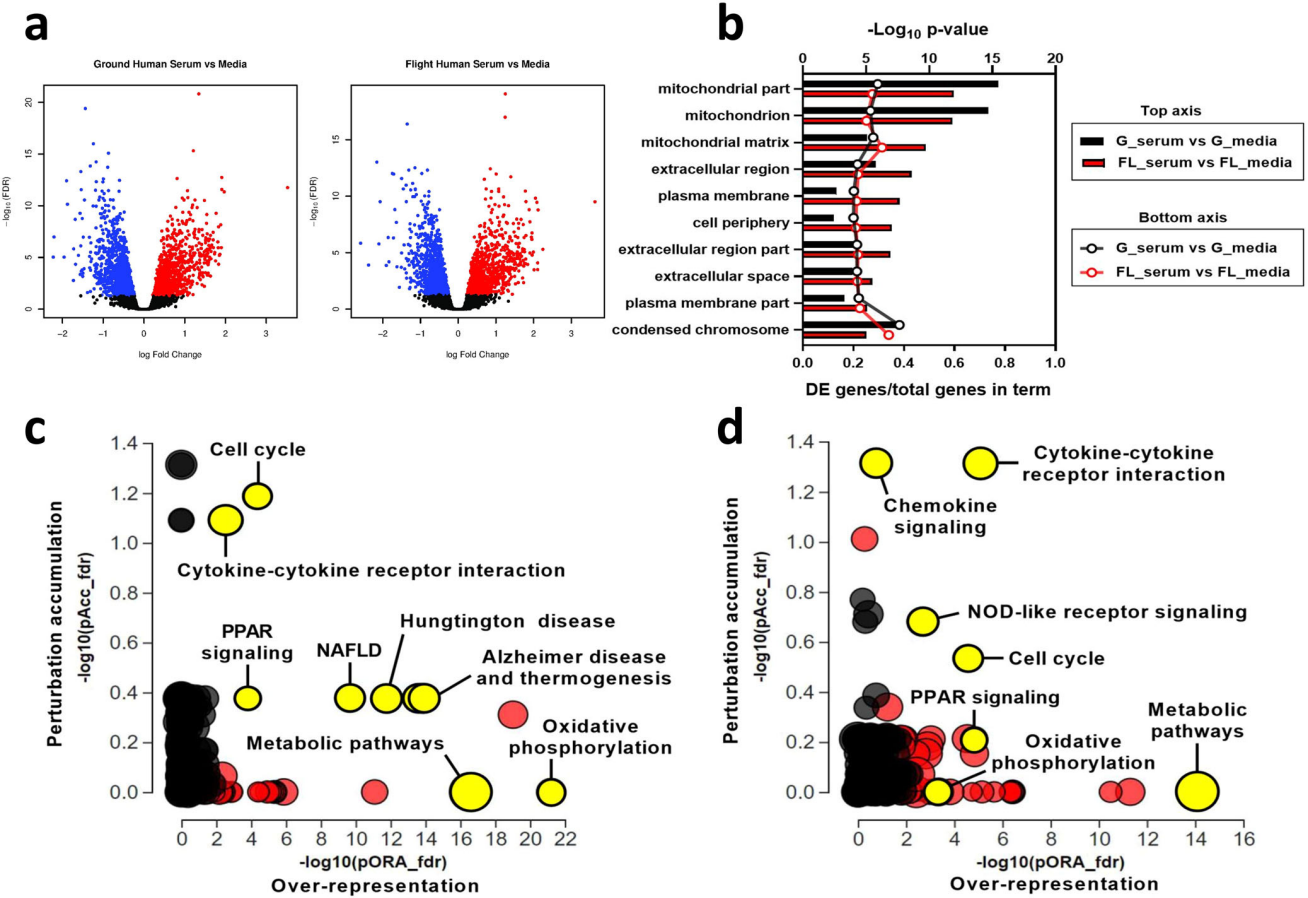
Serum promotes PTEC proliferation, alters cellular energetics and modifies the disposition of the extracellular environment. **a** Volcano plots depicting DE genes between serum and control treatments in the ground (left) and flight (right) conditions. **b** Gene ontology analysis of the set of DE genes showing enrichment in cellular component terms associated with mitochondria, extracellular space, and nucleus. Top axis displays FDR-adjusted *p*-value for the cellular component term, while the bottom axis displays the ratio of DE genes over the total number of genes within that specific term. **c** and **d** iPathwayGuide analysis plots of the FDR adjusted impact analysis *p*-value (pAcc) on the *y*-axis and over-representation p-value (pORA) on the *x*-axis for pathways identified as affected by serum treatment in ground (**c**) and flight (**d**) conditions. Black circles represent non-significant pathways, while red circles indicate significant pathways and yellow circles indicate significant pathways of interest. The size of the circle corresponds to the number of genes within the pathway.

To characterize the changes induced by serum exposure and identify condition-dependent responses, RNA from multiple replicates of control- or serum-treated PT-MPS was isolated and transcriptomic profiles were measured by RNA-seq. Exposure of PT-MPS to 2% normal human serum resulted in differential expression of 2389 and 2220 genes compared to control in the ground and flight conditions, respectively, based on a fold change of at least 1.1 at an adjusted *p*- value threshold of 0.05. In the ground condition, 1144 and 1245 genes were up- and down-regulated, respectively, whereas in the flight condition, 1108 and 1112 genes were up- and down-regulated, respectively (Fig. 3a). No genes were differentially expressed between (1) ground media vs. flight media, (2) ground serum vs. flight serum, or (3) (ground serum vs. ground media) vs. (flight serum vs. flight media), indicating that (1) flight alone did not impact PTEC gene expression, (2) the relative expression level for a given gene between the ground and flight serum-treated samples was similar, and (3) the flight condition did not affect the magnitude of change in expression of a gene between control treatment and serum treatments (i.e., the difference in differences).

To elucidate the functional networks regulated by serum exposure in PTECs, we performed Advaita gene ontology analyses and iPathwayGuide analyses on the genes differentially expressed between serum and control treatments in the ground and flight conditions. Gene ontology enrichment analysis showed over-representation of the set of DE genes in cellular component terms, such as mitochondrion (GO:0005739), plasma membrane (GO:0005886), extracellular space (GO:0005615), and condensed chromosome (GO:0000793) (Fig. 3b). While the false discovery rate (FDR) adjusted *p*-value calculated for each cellular component term was different between ground and flight, the number of DE genes within a given term was comparable suggesting the overall response to serum between ground and flight chips was similar (Fig. 3b). Advaita pathway analysis revealed that several cellular pathways were significantly affected by serum treatment in both the ground and flight conditions including cell cycle (ground: *p* = 3.19 × 10^–6^ and flight: *p* = 7.7 × 10^–6^), cytokine–cytokine receptor interaction (ground: *p* = 6.93 × 10^–6^ and flight: *p* = 1.12 × 10^–8^), chemokine signaling (ground: *p* = 0.0337 and flight: *p* = 0.0039), peroxisome proliferator-activated receptor (PPAR) signaling (ground: p = 1.49×10^–4^ and flight: *p* = 2.54 × 10^–5^), and metabolic pathways (ground: *p* = 3.96 × 10^–17^ and flight: 8.16 × 10^–15^) (Fig. 3c and d). Most of the genes within the cell cycle, cytokine–cytokine receptor interaction and chemokine signaling pathways were upregulated. Examination of the cell cycle pathway showed upregulation of genes that promote progression through the G1, S, G2, and M stages of the cell cycle (Supplementary Figs. 1 and 2). Several members of the CC chemokine, CXC chemokine, and interleukin families were upregulated in the chemokine signaling and cytokine-cytokine receptor interaction pathways (Supplementary Table 1). On the other hand, the PPAR signaling and metabolic pathways were downregulated. More specifically, genes within fatty acid metabolism (ground: *p* = 7.39 × 10^–6^ and flight: *p* = 3.79 × 10^–7^), tricarboxylic acid cycle (ground: *p* = 4.05 × 10^–3^ and flight: *p* = 4.09 × 10^–7^), and steroid biosynthesis (ground: *p* = 3.3 × 10^–5^ and flight: *p* = 1.9 × 10^–5^) pathways were downregulated (Supplementary Table 2). Non-alcoholic fatty liver disease, Alzheimer's disease, and Huntington disease pathways were affected only in the ground condition. Inspection of the DE genes within those pathways indicated that the statistical significance was largely driven by a group of mitochondrial genes associated with oxidative phosphorylation (Fig. 3c and d). Consistent with this observation, the oxidative phosphorylation pathway was far more impacted by 2% human serum treatment in the ground condition (*p* = 1.87 × 10^–24^, 63 DE genes) than in the flight condition (*p* = 2.5 × 10^–6^, 33 DE genes). Overall, these data suggest that serum exposure caused PTECs to activate a proliferative program, shift cellular bioenergetics, and promote a pro-inflammatory extracellular environment.

**Fig. 4:**
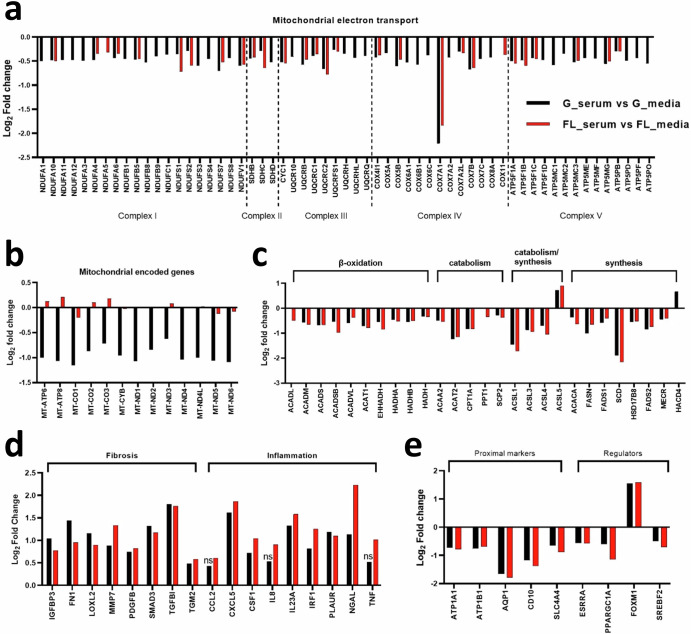
Serum induces metabolic reprogramming and a tissue remodeling gene signature in PTECs. Select genes that were differentially expressed after 48-hour 2% human serum in PTECs which function in **a** mitochondrial electron transport chain, **b** mitochondrial-encoded electron transport chain, **c** fatty acid metabolism, **d** fibrosis and inflammation, and **e** proximal tubule makers and transcriptional regulators. Data are presented as the log_2_-fold change in gene expression of serum samples relative to control samples in the ground condition (black bars) and the flight condition (red bars). Genes that were not statistically significant are noted by “ns”. G_serum ground serum, G_media ground control, FL_serum flight serum, FL_media flight control.

Next, we focused on gene-level changes to help delineate the biological consequence of exposure of PT-MPS to serum. First, we looked at metabolic reprogramming as it included the largest set of genes and was the most significantly impacted by serum treatment. Adenosine triphosphate (ATP) is a molecule that plays an important role in signal transduction (via being a substrate for kinases) and provides energy to drive a variety of cellular processes, including the transport of ions and solutes via ATP-binding cassette transporters such as the sodium–potassium-ATPase. PTECs generate the bulk of ATP through mitochondrial oxidative phosphorylation, wherein the transfer of electrons from nicotinamide adenine dinucleotide hydride (NADH) and dihydroflavin adenine dinucleotide (FADH_2_) to molecular oxygen (O_2_) through a series of protein complexes (complexes I–IV) in the mitochondrial inner membrane results in pumping of protons across the inner mitochondrial matrix membrane. This creates a transmembrane pH gradient that is subsequently utilized by complex V (or ATP synthase) to create ATP from adenosine diphosphate (ADP) and phosphate (Pi)^20^. The DE genes within the oxidative phosphorylation pathway were found to be involved in the mitochondrial electron transport chain, with the representation of all five of the major respiratory chain protein complexes (Fig. 4). A greater number of genes were identified in the ground condition compared to flight condition (58 vs. 27, respectively), though all DE genes in both conditions were downregulated by similar magnitudes suggesting that both ground and flight conditions had reduced mitochondrial respiration following serum treatment. The mitochondrion has its own genome which encodes thirteen proteins that participate in the electron transport chain^20,21^. Thirteen of those mitochondrial encoded genes were downregulated in the ground condition, but not the flight condition (Fig. 4b). The expression of four key factors that control mitochondrial gene transcription including RNA polymerase mitochondrial (*POLRMT*), transcription factor A mitochondrial (*TFAM*), transcription factor B2 mitochondrial (*TFB2M*), and transcription elongation factor mitochondrial (*TEFM*), were unchanged with serum treatment in both the flight and ground conditions (adj. *p*-value = 1).

To fuel the mitochondrial electron transport chain and oxidative phosphorylation, a steady source of reducing equivalents of NADH and FADH_2_ is required^20^. β-oxidation of fatty acids and intermediary metabolism in the tricarboxylic acid (TCA) cycle, each of which occurs in mitochondria, are the primary processes that generate FADH_2_ and NADH^20^. β-oxidation is the stepwise enzymatic process that shortens fatty acid chains by two carbon atoms, producing acetyl coenzyme A (acetyl-CoA), NADH, and FADH_2_^22^. Acetyl-CoA can subsequently be utilized in the TCA cycle, a series of chemical reactions that oxidize acetate (derived from acetyl-CoA) to ultimately produce GTP, NADH, FADH_2_, and carbon dioxide^20^.

Serum treatment of the PT-MPS significantly reduced the expression of a set of genes that function in β-oxidation, catabolism, and synthesis of fatty acids including carnitine palmitoyltransferase 1 (*CPT1A*), a transporter that is rate-limiting in fatty acid β-oxidation, and acetyl-CoA carboxylase alpha (*ACACA*) and fatty acid synthase (*FASN*), the rate-limiting enzymes in fatty acid biosynthesis (Fig. 4c). In addition, serum caused a modest, but broad downregulation of genes within the TCA cycle and solute carrier 25 (SLC25) family, that are mitochondrial membrane transporters for a variety of ions and metabolic intermediates (Supplementary Table 1). Expression of the lipogenic enzymes (*GPAT3, GPAT4, AGPAT1–5, DGAT1*, and *DGAT2*) was unchanged (data not shown) whereas only lipin 1 (*LPIN1)* was significantly reduced (Supplementary Table 2). The expression of genes that are targets of the sterol-regulatory element binding transcription factor 2 (*SREBF2*) was considerably repressed (Supplementary Table 2). Because SREBF2 activity is inhibited in the presence of high cellular cholesterol levels, repression of SREBF2 target genes indicated that serum treatment increased cytosolic cholesterol levels. The transcriptional repression of genes involved in fatty acid metabolism, cholesterol metabolism, and intermediary metabolism (TCA cycle) strongly indicated that serum treatment caused metabolic reprograming in PTECs.

Next, we evaluated genes that could be potential maladaptive effectors in the tubular response to protein challenge. Serum treatment induced the expression of genes that function in the extracellular space and are associated with tissue remodeling. This group of genes included extracellular matrix proteins (e.g., fibronectin 1 (*FN1*) and transforming growth factor beta induced (*TGFBI*)), growth factors (e.g., platelet derived growth factor beta (*PDGFB*)), transcription factors (e.g., mothers against decapentaplegic homolog 3 (*SMAD3*)), and extracellular matrix modifying enzymes (e.g., matrix metallopeptidase 7 (*MMP7*), lysyl oxidase like 2 (*LOXL2*), transglutaminase 2 (*TGM2*)) (Fig. 4d). It also induced pro-inflammatory molecules including chemokines (e.g., chemokine c-x-c motif chemokine ligand 5 (*CXCL5*)), cytokines (e.g., interleukin 8 (*IL8*), interleukin 23a (*IL23A*), tumor necrosis factor (*TNF*)), transcription factors (e.g., interferon regulatory factor 1 (*IRF1*)), and the lipocalin neutrophil gelatinase-associated lipocalin (*NGAL* or *LCN2*) (Fig. 4d). *IRF1*, *LCN2* (*NGAL*), *PLAUR*, *LOXL2*, *TGFBI*, and *SMAD3* are among the top 20 most significantly upregulated genes in PTECs following 2% human serum treatment (Supplementary Tables 3 and 4).

Because the loss of metabolic capacity and gain of pro-inflammatory and pro-fibrotic attributes could be detrimental to PTEC function, we next looked at whether the expression of proximal marker genes changed. Serum treatment caused a downregulation of several genes selectively expressed by PTECs in vivo including the water channel aquaporin 1 (*AQP1*) and the sodium potassium-transporting ATPase subunits alpha and beta (*ATP1B1* and *ATP1A1*) (Fig. 4e).

Concomitantly, there was downregulation of three key transcriptional regulators: peroxisome proliferator-activated receptor gamma coactivator 1-alpha (*PPARGC1A*), estrogen-related receptor alpha (*ESRRA*), and *SREBF2*, while forkhead box M1 (*FOXM1*) was induced (Fig. 4e). *ATP1B1* and *PPARGC1A* were among the top 20 downregulated genes in the flight condition (Supplementary Table 4). Advaita upstream regulator analysis identified both TNF (ground: *p* = 9.0 × 10^–3^ and flight: *p* = 2.4×10^–2^) and FOXM1 (ground: *p* = 2.71 × 10^–9^ and flight: *p* = 1.31 × 10^–8^) as transcription factors likely to have been activated by serum treatment based on the number of consistently observed DE genes and gene interactions (Table 2). Conversely, SREBF2 (ground: *p* = 5.86 × 10^–9^ and flight: *p* = 8.31 × 10^–9^) and PPARGC1A (ground: *p* = 1.2 × 10^–1^ and flight: *p* = 1.75 × 10^–2^) were predicted to have been inhibited by serum treatment (Table 2). The target genes of PPARGC1A include genes involved in mitochondrial oxidative phosphorylation (e.g., *CPT1A* and *EHHADHA*) as well as genes with regulatory roles (e.g., *ESRRA* and *SIRT3*), while those of FOXM1 tend to be related to cell proliferation (e.g., *CCNA1* and *CCNB1*) and DNA damage response (e.g., *RAD51* and *RAD54*).

**Table 2:** Upstream regulators of the transcriptional response of PTECs to 2% human serum

Upstream regulator	Directional change	Ground serum vs. Ground media			Flight serum vs. Flight media		
		DE targets (±)/ DE targets	*p*-value	FDR *p*-value	DE targets (±)/ DE targets	*p*-value	FDR *p*-value
TNF	Activated	32/45	6.15E−04	9.00E−03	31/42	1.00E−03	2.40E−02
FOXM1	Activated	18/18	1.27E−10	2.71E−09	18/19	5.45E−10	1.31E−08
SREBF2	Inhibited	15/15	2.99E−12	5.86E−09	15/15	3.76E−12	8.31E−09
PPARGC1A	Inhibited	7/8	1.00E−03	**1.20E−01**	8/9	1.75E−04	1.40E−02
PPARA	Inhibited	12/16	8.00E−03	**4.94E−01**	16/20	6.50E−05	8.00E−03
RXRA	Inhibited	10/12	5.00E−03	**3.57E−01**	16/20	5.26E−06	1.00E−03

Proteins predicted to have been inhibited or activated by 2% human serum treatment in PTECs in ground and flight conditions based on Advaita upstream regulator analysis. Upstream regulators with non-significant *p*-values are noted by bold text. For each upstream regulator, the predicted directional change in activity (activation or inhibition) with 2% human serum treatment is shown. The DE targets (±)/DE targets column depicts the number of target genes with a directional change in expression consistent with the predicted change in upstream regulator activity over the total number of differentially expressed target genes for that upstream regulator. The unadjusted *p*-value and FDR-adjusted *p*-value are presented for each upstream regulator.

### PT-MPS biomarker responses to 2% human serum in flight and ground conditions

**Fig. 5:**
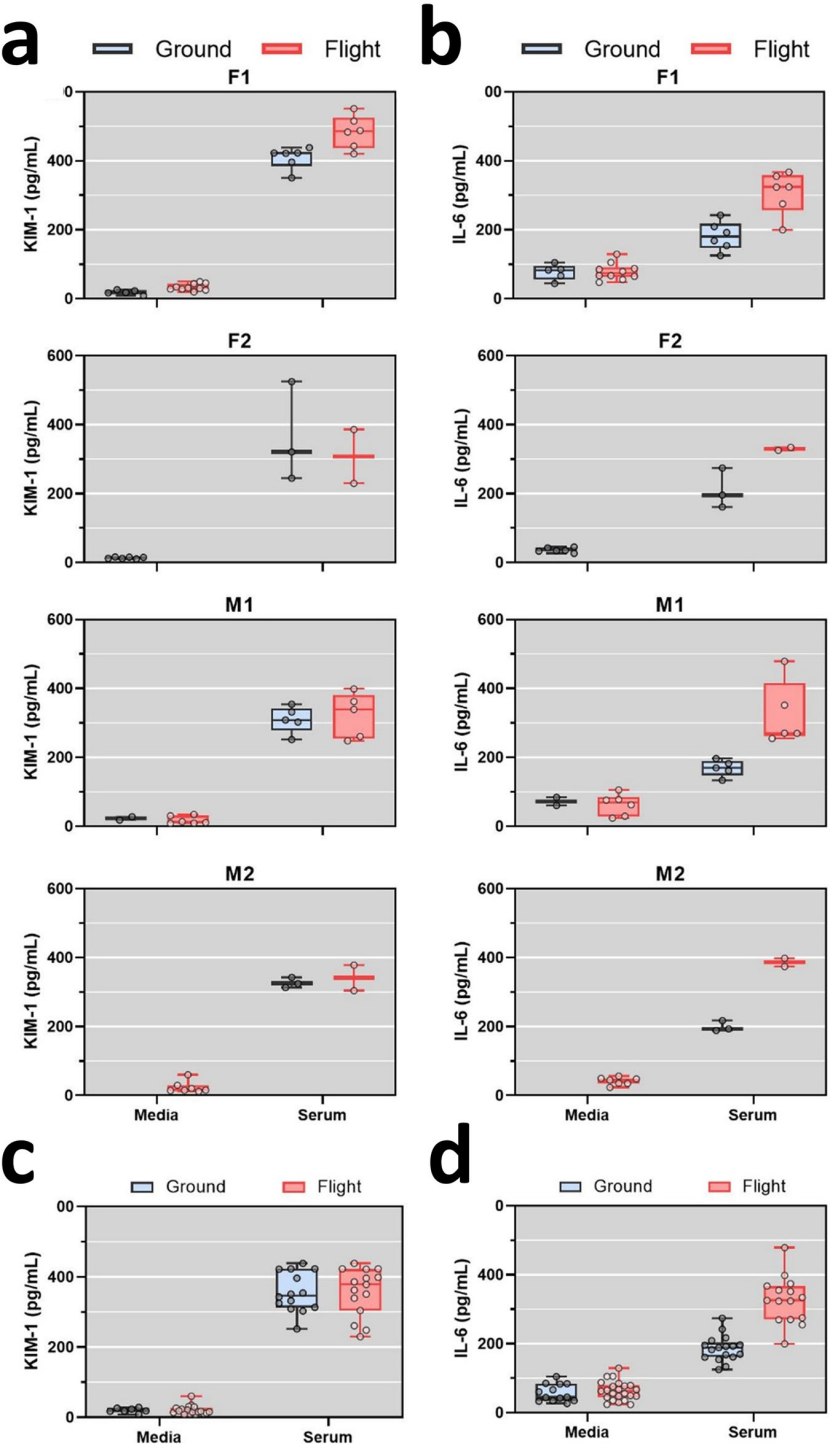
Serum-induced secretion of KIM-1 and IL-6. **a** and **b** Quantification of KIM-1 (**a**) or IL-6 (**b**) levels in PT-MPS effluents after 48 h of treatment with media control or 2% human serum in ground (black) and flight conditions (red) from two female donors (F1 and F2) and two male donors (M1 and M2). **c** KIM-1 levels of all four donors in each treatment and condition. KIM-1 secretion was significantly increased by treatment with serum (*p* < 0.0001; one-way ANOVA). **d** IL-6 levels of all four donors in each treatment and condition. IL-6 secretion was significantly increased by serum treatment in ground and flight (*p* < 0.0001; one-way ANOVA).

To validate the observations that 2% human serum appeared to promote transcription of cellular proliferation and induce proinflammatory genes, we quantified KIM-1 and IL-6 from device effluents. The magnitude of 2% human serum-induced secretion of KIM-1 and IL-6 varied by the donor but was consistently increased relative to media control (Fig. 5a and b). Serum treatment significantly increased KIM-1 secretion relative to media control for both ground (20.9-fold, *p* < 0.0001) and flight conditions (14.5-fold, *p* < 0.0001) (Fig. 5c). There was no difference in serum-induced secretion of KIM-1 between ground and flight. IL-6 secretion was significantly increased by serum treatment relative to media control in both grounds (3.3-fold, *p* = 0.0004) and flight conditions (5.2-fold, *p* < 0.0001) (Fig. 5d). The difference in IL-6 change from media control to serum between the flight and ground condition was not statistically different (*p* = 0.073, linear mixed effects model) suggesting that there was no interaction between microgravity and serum exposure on IL-6 secretion.

### Transcriptional response of PTECs to vitamin D in ground and flight conditions

**Fig. 6:**
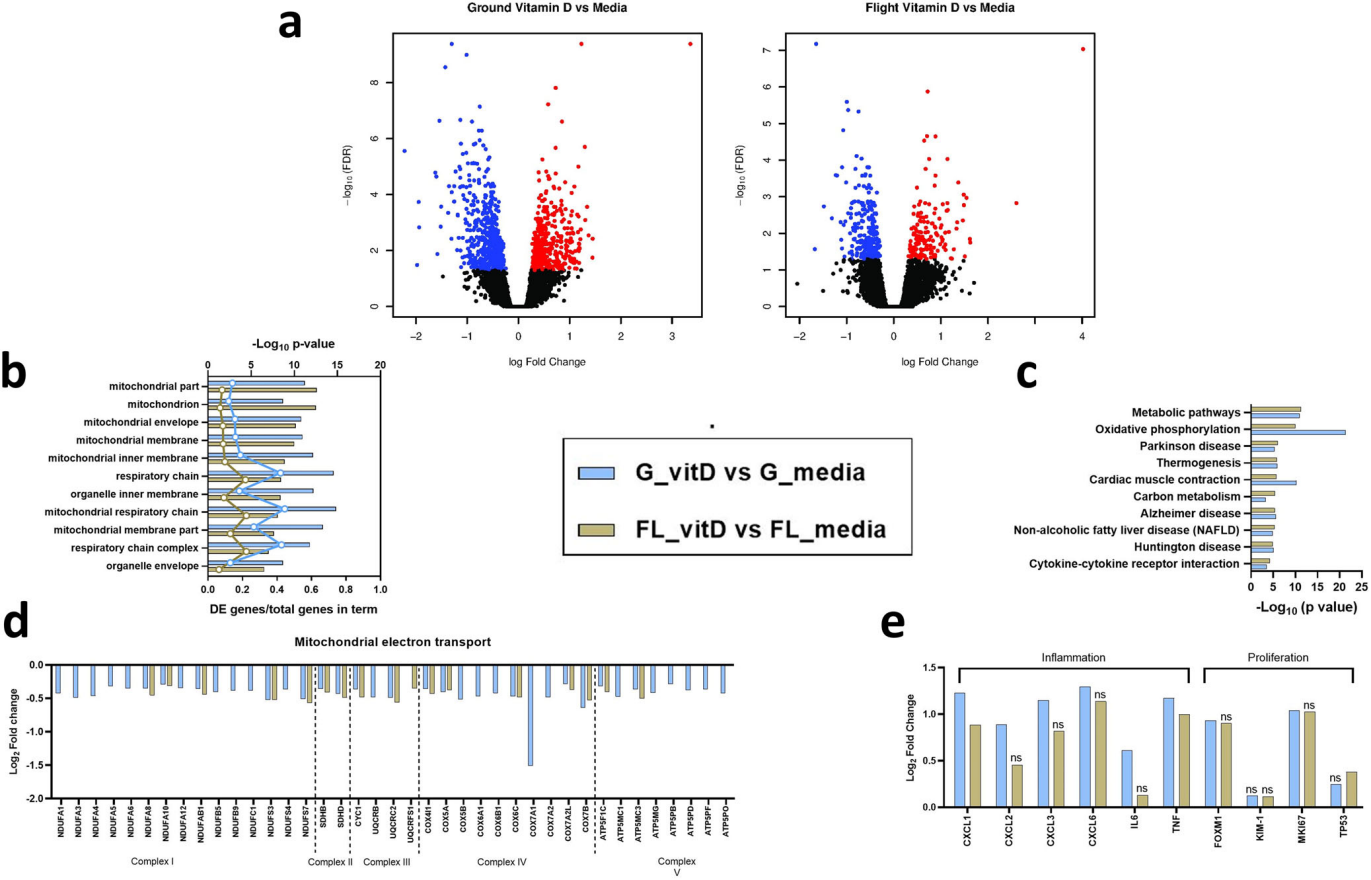
Vitamin D represses mitochondrial gene expression and induces chemokines in PTECs. **a** Volcano plots depicting DE genes between 25(OH)D_3_ and control treatments in the ground (left) and flight (right) conditions. **b** Gene ontology analysis of the set of DE genes showing enrichment of mitochondrial terms. Top axis displays the FDR-adjusted *p*-value for the cellular component term, while the bottom axis displays the ratio of DE genes over the total number of genes within that specific term. **c** iPathwayGuide analysis of the pathways significantly impacted by 25(OH)D_3_ treatment in ground and flight conditions. **d** Plot of genes differentially expressed in mitochondrial electron transport. **e** Selected genes involved in inflammation and cellular proliferation. The bars represent the comparison of vitamin D (25(OH)D_3_) and control (media) treated PT-MPS for the ground condition (blue bars) and the flight condition (gold bars). Genes that were not statistically significant are noted by “ns”.

To characterize the changes induced by vitamin D exposure and identify condition-dependent responses, RNA from multiple replicates of control- or 25(OH)D_3_-treated PT-MPS was isolated and transcriptomic profiles were measured by RNA-seq. Comparing the differentially expressed (DE) genes revealed 598 DE and 147 DE in the ground and flight groups, respectively (Fig. 6a). In each condition roughly half the genes were upregulated, and half were downregulated.

Gene ontology enrichment analysis revealed over-representation of the set of DE genes in cellular component terms such as mitochondrion (GO:0005739) and mitochondrial respiratory chain (GO:0005746) (Fig. 6b). The number of DE genes within each term varied by condition with the ground condition having a greater number DE in each term. Advaita iPathwayGuide analysis showed the pathways most affected by vitamin D treatment were metabolic pathways, oxidative phosphorylation, and cytokine–cytokine receptor interaction (Fig. 6c). Oxidative phosphorylation was more affected by vitamin D treatment on ground (*p* = 1.43 × 10^–19^, 42 DE genes) than in flight (*p* = 1.22 × 10^–8^, 19 DE genes). Consistent with this observation, more genes within the electron transport chain were downregulated in ground than in flight (Fig. 6d). Vitamin D treatment induced several members of the c-x-c motif ligand family in both conditions including *CXCL1*, *CXCL2*, *CXCL3* and *CXCL6*, while the cytokine interleukin 6 (*IL6*) was only induced with 25(OH)D_3_ treatment for the ground condition (Fig. 6e). The proliferation-associated genes *FOXM1* and marker of proliferation Ki67 (*MKI67*) were only significantly upregulated in the ground condition (Fig. 6e).

### Impact of microgravity on PTEC metabolism of vitamin D

**Fig. 7:**
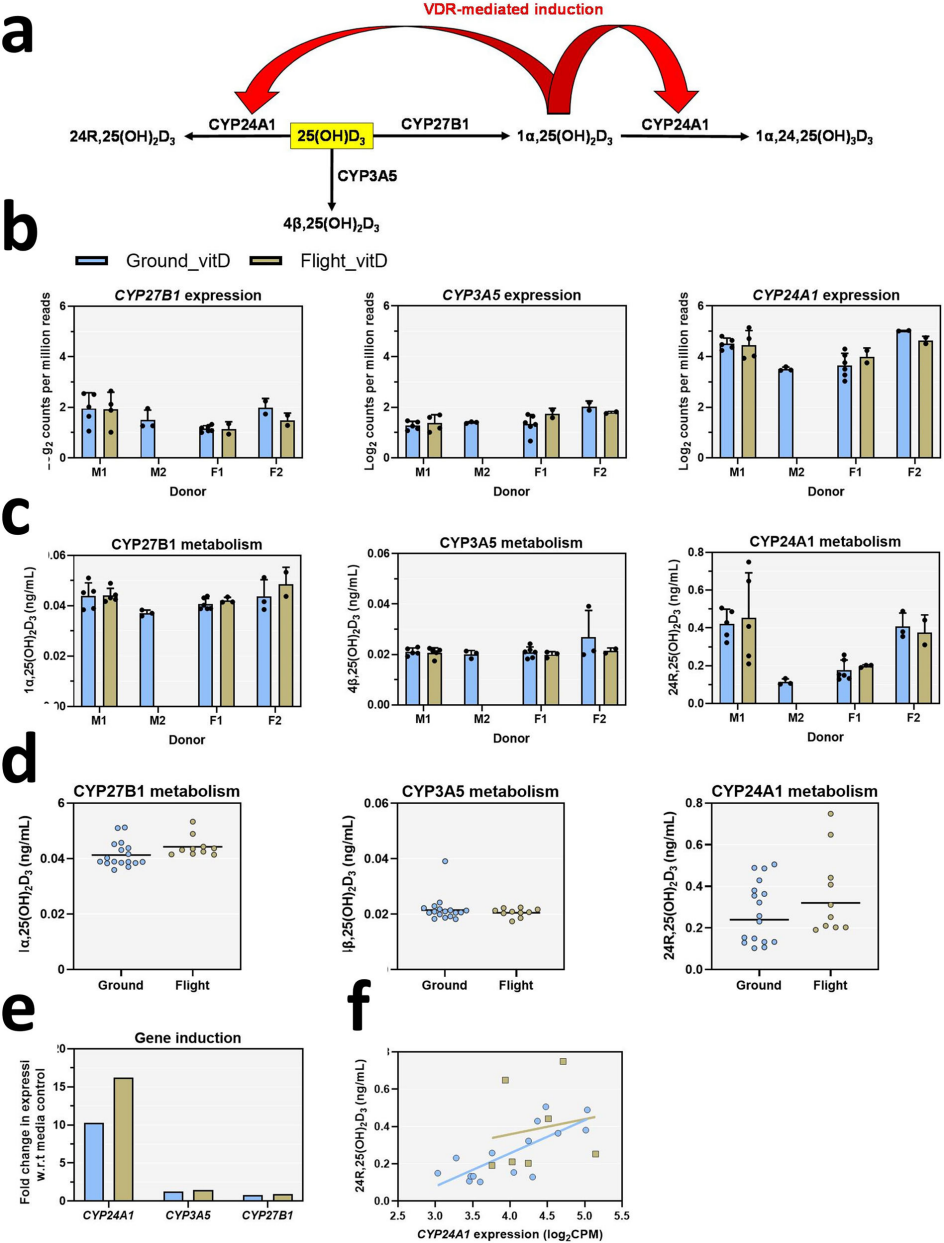
Microgravity does not alter the metabolism of vitamin D or transcriptional regulation of *CYP24A1.* **a** Scheme of vitamin D metabolism and feedback regulation of *CYP24A1* via the VDR in PTECs. 25(OH)D_3_ undergoes multiple metabolic reactions within PTECs including bioactivation to 1α,25(OH)_2_D_3_ via CYP27B1 as well as inactivation through CYP24A1 mediated conversion to 24R,25(OH)_2_D_3_ and CYP3A5 mediated conversion to 4β,25(OH)_2_D_3_. 1α,25(OH)_2_D_3_ undergoes sequential metabolism by CYP24A1 to 1α,24R,25(OH)_3_D_3_, however, this analyte was not quantified in this assay. 1α,25(OH)_2_D_3_ agonizes the VDR leading to induction of *CYP24A1*. **b** Plots of log_2_ counts per million reads of vitamin D metabolizing cytochrome P450 enzymes. Data are presented as mean±s.d. **c** Levels of vitamin D metabolites in device effluents formed from each P450 pathway separated by donor and condition. Data are presented as mean ± s.d. **d** Average metabolite concentrations of 1α,25(OH)_2_D_3_, 4β,25(OH)_2_D_3_, and 24R,25(OH)_2_D_3_ in ground and flight. **e** Fold-change in *CYP24A1*, *CYP3A5*, and *CYP27B1* expression after 48-h vitamin D treatment relative to media controls in ground and flight conditions. **f** Plot of 24R,25(OH)_2_D_3_ effluent levels compared to the corresponding expression of *CYP24A1* in that sample. *CYP24A1* expression was correlated with levels of 24R,25(OH)_2_D_3_ in ground samples (*r* = 0.77, *p* = 0.0008) but not flight samples (*r* = 0.17, *p* = 0.71). Ground condition samples are represented by blue bars/symbols while flight condition samples are represented by gold bars/symbols.

25(OH)D_3_ undergoes multiple metabolic reactions within PTECs including bioactivation to 1α,25(OH)_2_D_3_ via CYP27B1 as well as inactivation through CYP24A1 mediated conversion to 24R,25(OH)_2_D_3_ and CYP3A5 mediated conversion to 4β,25(OH)_2_D_3_ (Fig. 7a) To evaluate the impact of microgravity on PTEC metabolism of 25(OH)D_3_, we quantified 25(OH)D_3_ and its primary metabolites 1α,25-dihydroxy vitamin D_3_, 4β,25-dihydroxy vitamin D_3_, and 24R,25-dihydroxy vitamin D_3_ in the device effluents. Expression of *CYP3A5*, *CYP24A1*, and *CYP27B1* was detected in all samples (Fig. 7b). Formation of 1α,25(OH)_2_D_3_ and 4β,25(OH)_2_D_3_ was consistent across donors, whereas formation of 24R,25(OH)_2_D_3_ varied by donor (Fig. 7c). Formation of 1α,25(OH)_2_D_3_ (*p* = 0.1036), 4β,25(OH)_2_D_3_ (*p* = 0.4451), and 24R,25(OH)_2_D_3_ (*p* = 0.2228) did not differ between ground and flight (Fig. 7d). Consistent with the formation of 1α,25(OH)_2_D_3_ and agonism of the VDR, the expression of *CYP24A1* but not *CYP3A5* or *CYP27B1* was significantly higher in vitamin D-treated samples than in media controls for both ground and flight conditions (Fig. 7e). The expression of *CYP24A1* was correlated with the formation of 24R,25(OH)_2_D_3_ in ground samples (*r* = 0.77, *p* = 0.008) but not flight samples (*r* = 0.17, *p* = 0.715) (Fig. 7f).

### PT-MPS biomarker responses to vitamin D in flight and ground conditions

**Fig. 8:**
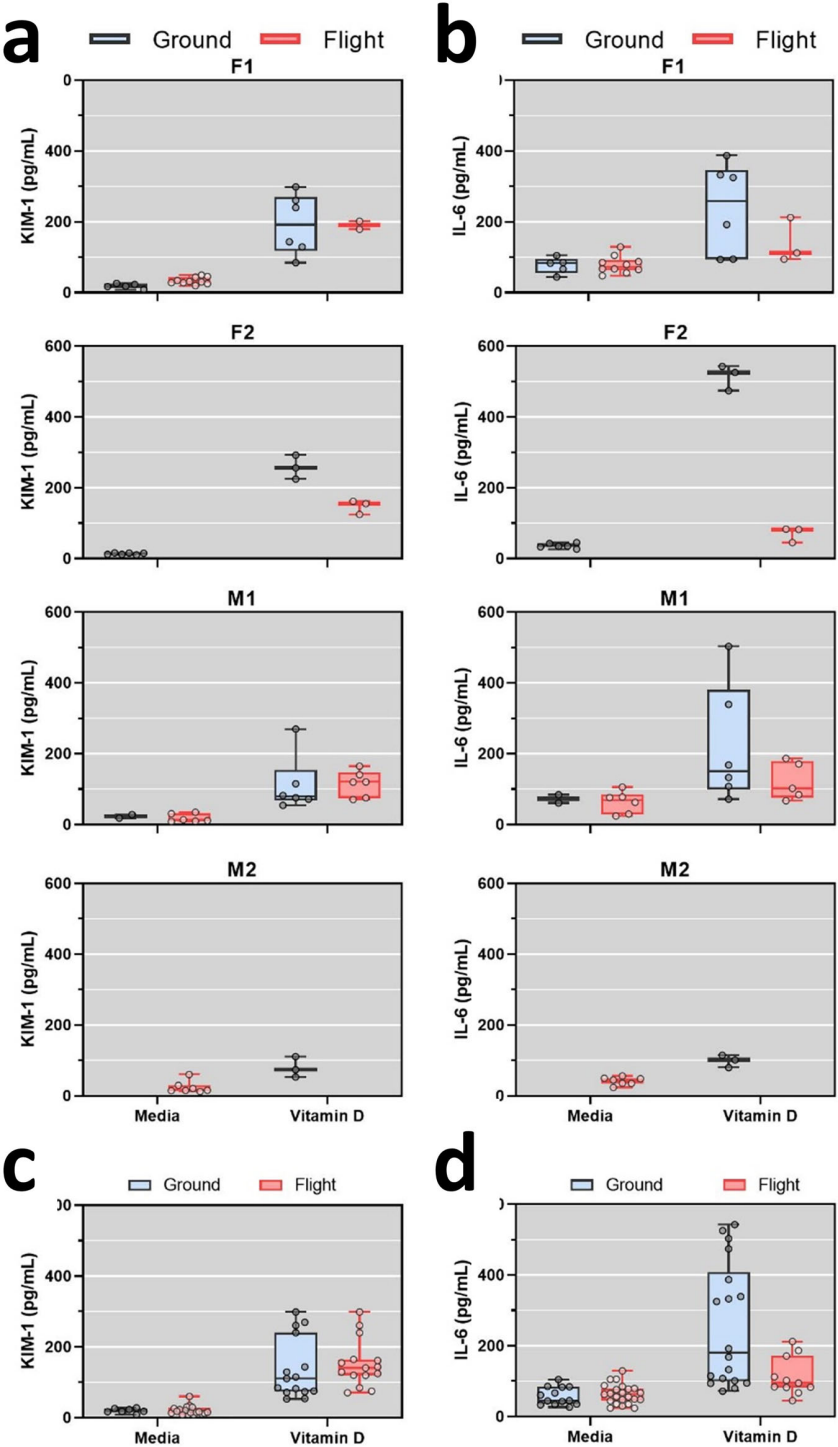
Vitamin D-induced secretion of KIM-1 and IL-6. Quantification of KIM-1 (**a**) or IL-6 (**b**) levels in PT-MPS effluents after 48 h of treatment with media control or 3 µM vitamin D binding protein (DBP) + 1.5 µM vitamin D (25(OH)D_3_) in the ground (black) and flight conditions (red) from two female donors (F1 and F2) and two male donors (M1 and M2). **c** KIM-1 secretion of all four pooled donors was significantly increased by treatment with Vitamin D (*p* < 0.0001; one-way ANOVA). **d** IL-6 secretion of all four pooled donors was significantly increased by Vitamin D treatment in the ground (*p* < 0.0001; one-way ANOVA) and flight (*p* = 0.0018; one-way ANOVA). In addition, when comparing levels of Il-6 of all four pooled donors in vitamin D-treated PT-MPS between ground and flight, the levels in flight were significantly lower in comparison to the ground (*p*-value = 0.001; one-way ANOVA).

To assess whether treatment with vitamin D resulted in effluent biomarkers similar to PT-MPS, we measured KIM-1 and IL-6 in flight and ground samples. Comparison of samples for both biomarkers demonstrated consistent increases for all four donors for flight and ground, although interpretation is limited by sample availability (Fig. 8a and b). Vitamin D treatment significantly increased KIM-1 secretion relative to media control for both ground (9.2-fold, *p* < 0.0001) and flight conditions (5.2-fold, *p* < 0.0001) (Fig. 8c). IL-6 secretion was significantly increased by Vitamin D treatment in ground (*p* < 0.0001) and flight (*p* = 0.0018) (Fig. 8d). In addition, when comparing levels of IL-6 in vitamin D-treated PT-MPS between ground and flight, the levels in flight were significantly lower in comparison to ground (*p*-value = 0.001).

## Discussion

Plasma levels of 1α,25(OH)_2_D_3_ have been shown to decrease over time in astronauts on flight. This phenomenon could be due to several known factors, including (1) changes in hydrostatic pressure that drive the movement of water and protein from the intravascular space to intracellular and interstitial compartments resulting in hemodilution^23^ or (2) a partial decoupling of the renin-angiotensin-aldosterone-vasopressin system due to hypercalciuria secondary to bone mineral loss on orbit^24^. Our team explored the hypothesis that microgravity-induced changes in PTEC-mediated metabolism of Vitamin D might also contribute to the observed decline in plasma levels of 1α,25(OH)_2_D_3_.

In both the ground condition and flight condition, PTECs treated with 25(OH)D_3_ generated 1α,25(OH)_2_D_3_, 4β,25(OH)_2_D_3_, and 24R,25(OH)_2_D_3_, the primary metabolites of CYP27B1, CYP3A5, and CYP24A1, respectively (Fig. 7). The levels of these metabolites did not differ between ground and flight conditions. Induction of *CYP24A1*, a canonical target gene of the VDR, was robust indicating that the feedback mechanism within PTECs was intact and did not differ between ground and flight conditions (Fig. 7). We conclude that microgravity did not alter the metabolic activity of CYP27B1, CYP24A1, or CYP3A5, nor did it significantly alter the inducibility of *CYP24A1*, a feedback mechanism which helps to tightly regulate plasma levels of 1α,25(OH)_2_D_3_. Regarding effluent biomarker responses to 25(OH)D_3_, we observed increases in both KIM-1 and Il-6, for both flight and ground groups (Fig. 8). While the levels were generally lower than what was observed with 2% normal human serum, it is still interesting to note given these biomarkers are typically associated with tubular injury. Also of note are the differences between flight and ground responses for Il-6, where levels were significantly lower in flight, suggesting attenuated responses that are congruent with the lower number of DEGs 598 versus 147 (Figs. 6a and 8d). While this is an interesting observation, it is difficult to speculate why this is occurring over this short time and should be re-evaluated for longer periods of time in microgravity, given the average time of 6 months astronauts are stationed onboard the ISS.

The metabolite 1α,24R,25-trihydroxy vitamin D3 (1α,24R,25 (OH)_3_D_3_), which is generated by CYP27B1-mediated metabolism of 1α,25(OH)_2_D_3_, was not measured in our assay. Therefore, it is unclear whether the reason for the relatively low levels of 1α,25(OH)_2_D_3_ was poor formation from 25(OH)D_3_ or rapid elimination to 1α,24R,25(OH)_3_D_3_. The levels of 25(OH)D_3_ (~750 ng/mL) used in our study were supraphysiological and far exceeded those of 1α,25(OH)_2_D_3_ (~0.04 ng/mL). The average ratio of 25(OH)D_3_:1α,25(OH)_2_D_3_ in human plasma is 1:500, whereas in this study it was 1:18,750^25^. While 1α,25(OH)_2_D_3_ is the most potent vitamin D metabolite binding to the VDR, 25(OH)D_3_ can compete with 1α,25(OH)_2_D_3_ for binding of intestinal chromatin homogenates when administered at concentrations 150-fold higher than that of 1α,25(OH)_2_D_3_^26^. Similarly, 25(OH)D_3_ stimulates calcium transport, a marker of VDR activity, in the perfused intestine when administered at levels 200-times that of 1α,25(OH)_2_D_3_^26^. In vitro, there is strong evidence that 25(OH)D3 can activate the VDR^27,28^. Thus, both 25(OH)D_3_ and 1α,25(OH)_2_D_3_ can elicit VDR responses if the ratio of 25(OH)D_3_:1α,25(OH)_2_D_3_ is >200. Consequently, as the actual intracellular levels of 1α,25(OH)2D_3_ were unknown in this study, it is unclear whether activation of the VDR and induction of *CYP24A1* was triggered by 25(OH)D_3_ or 1α,25(OH)_2_D3. Nevertheless, we can conclude that microgravity did not appear to affect the metabolism of 25(OH)D_3_ via CYP27B1, CYP3A5, or CYP24A1in this study it was 1:18,750^25^. While 1α,25(OH)2D3 is the most potent vitamin D metabolite binding to the VDR, 25(OH)D3 can compete with 1α,25(OH)2D3 for binding of intestinal chromatin homogenates when administered at concentrations 150-fold higher than that of 1α,25(OH)_2_D_3_^26^. Similarly, 25(OH)D_3_ stimulates calcium transport, a marker of VDR activity, in the perfused intestine when administered at levels 200-times that of 1α,25(OH)_2_D_3_^26^^.^ In vitro, there is strong evidence that 25(OH)D_3_ can activate the VDR^27,28^. Thus, both 25(OH)D_3_ and 1α,25(OH)_2_D_3_ can elicit VDR responses if the ratio of 25(OH)D_3_:1α,25(OH)_2_D_3_ is >200. Consequently, as the actual intracellular levels of 1α,25(OH)_2_D_3_ were unknown in this study, it is unclear whether activation of the VDR and induction of *CYP24A1* was triggered by 25(OH)D_3_ or 1α,25(OH)_2_D_3_. Nevertheless, we can conclude that microgravity did not appear to affect the metabolism of 25(OH)D_3_ via CYP27B1, CYP3A5, or CYP24A1.

We also investigated the possibility that microgravity could affect the response of PTECs to proteinuria^29,30^; we tested whether the biological response was altered in flight compared to ground condition by treating the PTECs with 2% normal human serum. In both ground and flight conditions, pathway analysis revealed serum treatment-induced genes associated with proliferation, inflammation, and reorganization of the extracellular matrix environment, with concomitant downregulation of metabolic and biosynthetic pathways. The transcriptional and protein-level response of PTECs to 2% normal human serum did not differ between ground and flight conditions. While there was no condition-dependent response of PTECs to 2% human serum treatment, the observed transcriptional responses suggest PTECs have the potential to promote renal inflammation and fibrosis during proteinuria.

One mechanism by which PTECs acquire a proinflammatory phenotype is through cell cycle arrest at either the G1/S or G2/M phases of the cell cycle. While there are no definitive transcriptional markers of cell cycle arrest, we observed the induction of genes involved in cell cycle arrest. For example, SMAD3 was the first and second most significantly induced gene by serum treatment in ground and flight, respectively (Supplementary Tables 3 and 4). SMAD3 is strongly associated with renal fibrosis as SMAD3 knockout prevents fibrosis in mouse models of UUO, diabetic nephropathy, hypertensive nephropathy, and chronic aristolochic acid nephropathy^31–34^. A potential mechanism by which SMAD3 contributes to renal fibrosis is the promotion of cell cycle arrest. For example, SMAD3 contributes to c-reactive protein-mediated G1/S cell cycle arrest in a mouse model of IRI and in the human kidney 2 (HK-2) cells^35^. Arrest of proximal tubule cells in the G2/M phase has also been implicated in the acquisition of a proinflammatory secretory phenotype in IRI, UUO, and aristolochic acid nephropathy mouse models of AKI^36^. Arrest in the G2/M phase would be expected to be associated with higher levels of DNA damage response transcripts. In our data, we observed that several DNA damage response transcripts were induced including *RAD51*, *RAD54*, and *BRCA1*. However, *RAD51*, *RAD54*, and *BRCA1* have also been shown to be downstream targets of FOXM1 during epithelial repair after IRI^37^. Whether serum treatment increases the proportion of PTECs arrested at either the G1/S or G2/M stages should be investigated in future studies.

In summary, we demonstrated that microgravity neither altered PTEC metabolism of vitamin D nor did it induce a unique response of PTECs to human serum. The decline in the plasma levels of 1α,25(OH)_2_D_3_ in astronauts in flight appears to be independent of a change in renal expression of vitamin D metabolizing enzymes. Future efforts should focus on delineating the role of PTH and serum calcium on PTEC metabolism of vitamin D. The overall response of PTECs to serum challenge is congruent with the maladaptive repair response in vivo in which a failure of PTECs to re-differentiate after tubular injury is associated with tissue inflammation and fibrosis. The factors regulating PTEC differentiation status during proteinuric and disease states should further be elucidated, and their potential as novel therapeutic targets for treating and preventing renal inflammation and fibrosis should be investigated.

## Methods

### Cell culture

Deidentified human cortical kidney samples were collected through the Northwest Biotrust at the University of Washington Medical Center with local IRB approval (UW IRB Study 1297). Primary human proximal tubule epithelial cells were isolated by mechanical and enzymatic dissociation and cultured as previously described^38,39^. Serum-free tubular cell cultures were maintained in PTEC maintenance media consisting of DMEM/F12 (Gibco, 11330-032) supplemented with 1× insulin-transferrin-selenium-sodium pyruvate (ITS-A, Gibco, 51300044), 50 nM hydrocortisone (Sigma, H6909), and 1× Antibiotic–Antimycotic (Gibco, 15240062). Upon reaching 70–80% confluence, PTECs were passaged by enzymatic digestion with 0.05% trypsin EDTA (Gibco, 25200056) and manual cell scraping to obtain a single-cell suspension that was subsequently neutralized with defined trypsin inhibitor (Gibco, R007100). Cells were pelleted by centrifugation at 200 × *g* for 6 min, resuspended in maintenance media, and plated in cell culture-treated flasks at >30% confluency. PTECs were used at passage number 2–3 from all donors in these experiments.

### Microphysiological devices

Triplex microfluidic devices were purchased from Nortis, Inc. (Bothell, WA) and prepared as previously described^40^. Triplex microfluidic devices contain three fluidic circuits, which enable the generation of three PTEC tubules on a single device that can be continuously perfused with media.

### Maintenance, treatment, and fixation of PT-MPS

The BioServe perfusion platform was developed to house three Triplex devices in a self-contained, hermetically sealed system to meet the levels of containment required by NASA and reduce the space required to perfuse the Triplex devices. A flow rate of 0.5 µL/min was used for cell maintenance and treatment. The treatment conditions were control (PTEC maintenance media), vitamin D, or 2% human serum.^42^ To prepare 2% human serum treatment media, normal human serum (Valley Biomedical, HS1021) was diluted in PTEC maintenance media to a final concentration of 2%. Vitamin D treatment media consisted of PTEC maintenance media supplemented with 1.5 µM 25(OH)D_3_ (Toronto Research Chemicals, C125700) and 3 µM DBP (Athens Research, 16-16-070307). To prepare vitamin D treatment media, stock 25(OH)D_3_ was solubilized with molecular biology grade ethanol to 5 mM. DBP was reconstituted to 3 µM in PTEC maintenance media to create PTEC-DBP media. 25(OH)D_3_ was then diluted into PTEC-DBP media to 1.5 µM. Vitamin D media was allowed to equilibrate at room temperature for 30 min prior to filling the treatment cassette to ensure binding of 25(OH)D_3_ to DBP. The final concentration of ethanol in the vitamin D treatment media was 0.02%. In order to preserve the tubules at the end of treatment for analyses, the devices were fixed for 2 ^h^ with either 10% neutral buffered formalin (Thermo, 5725) or RNALater (ThermoFisher, AM7024) at a flow rate of 10 µL/min.

### RNA isolation, sequencing, and analysis

RNA was isolated from devices fixed with RNALater by injecting 100 µL of RLT lysis buffer (Qiagen, 79216) into the injection port using a 1 mL syringe outfitted with a 22-gauge needle. The cell lysate was collected at the outlet, 400 µL of RLT lysis buffer was added to each tube, and the samples were stored at −80 °C until extraction. RNA was extracted using the RNeasy Micro Kit (Qiagen, 74004) and converted to cDNA with the SMART-Seq v4 Ultra Low Input RNA Kit (Takara, 634891). Sequencing libraries were constructed using the SMARTer ThruPlex DNA-Seq Kit (Takara, R400676) and sequenced on a NovaSeq 6000 instrument (Illumina, San Diego, CA). Sequencing reads were aligned to GRCh38.p12 with reference transcriptome GENCODE human release 30 (with additional ERCC spike-in sequences) using STAR (v2.6.1d).

### Statistical methods and model fitting

Prior to fitting models, we excluded genes that are expressed at consistently low levels across all samples^41^. Prior to filtering, we had data for 58,870 genes, and after filtering, we had data for 14094 genes. The trimmed mean of *M*-values (TMM) normalization method was conducted^42^. We used the voom method from the Bioconductor limma package, which estimates the mean-variance relationship of the log-counts per million (logCPM), and generates a precision weight for each observation and enters these into the limma analysis pipeline^43^. A small positive value was added to each raw count to avoid taking the logarithm of zero, and log CPM can be interpreted as normalized count data by the corresponding total sample counts (in millions). We used the linear mixed model approach, fitting the condition_treatment as the fixed effect and the donor as the random effect by estimating the within-donor correlation^44^. We then fit a linear model with condition_treatment and incorporated the within-donor correlation (corr = 0.3). Since not all donors received all the treatments under each condition, the mixed model approach provides more statistical power for the unbalanced design. Both observation level and sample-specific weights were used, which enabled up or down-weighting of individual samples. This allowed us to keep all samples in the analysis and minimized the need to make decisions about removing possible outlier samples from consideration. The approach of using observation level and sample-specific weights has been shown to increase power in both real and simulated studies^45^.

We selected genes based on a 1.1-fold or greater difference in expression and a false discovery rate (FDR) of 5%. Rather than using a post-hoc fold-change filtering criterion, we used the TREAT function from limma, which incorporates the fold-change into the statistic, meaning that instead of testing for genes that have fold-changes different from zero (H_0_:*β* = 0 versus HA:*β* ≠ 0), we tested whether the fold-change was greater than 1.1-fold in absolute value (H_0_:|*β* | ≤1.1 versus HA:|*β* | >1.1)^46^.

### Gene ontology and iPathwayGuide

Advaita iPathwayGuide scores pathways using the Impact Analysis method which considers two types of evidence (1) over-representation of DE genes in a given pathway relative to random chance (pORA) and (2) the perturbation of the pathway computed by taking into account factors such as the magnitude of each gene’s expression change, position within the pathway, and gene interactions (pAcc). In gene ontology (GO) analysis, the number of DE genes annotated to a term was compared with the number of DE genes expected by chance. Pathways and GO terms were determined to be significant at a false-discovery rate < 0.05.

### Quantification of IL-6 and KIM-1 by ELISA

The DuoSet® line of ELISAs from R&D Systems (Minneapolis, MN) was used to quantify the protein levels of IL-6 and KIM-1 (HAVCR1) from device effluents according to the manufacturer’s instructions. The levels of IL-6 and KIM-1, in 2% human serum were below the limit of detection. Samples were assayed in technical duplicates.

### Statistical analysis of IL-6 and KIM-1 effluent biomarkers

To investigate whether there exists an interaction between condition and treatment groups, specifically to determine if changes in KIM- 1 or IL-6 levels among treatment groups (media control, 2% human serum, and vitamin D) vary in flight versus ground conditions, we employed a linear mixed effect model. This model incorporates the treatment group and condition as fixed effects and includes their interactions, with the donor serving as the random effect in a random intercept model. Prior to fitting the model, concentrations of KIM-1 and IL-6 were log2-transformed. The analysis was conducted using the lme function from the nlme package in R.

### Vitamin D analysis

Stock solutions and standard curves were prepared and described previously^47^. PTEC maintenance media containing 3 µM DBP (PTEC-DBP media) was used as a blank matrix. Quality control samples were prepared by diluting 1α,25(OH)_2_D_3_, 4β,25(OH)_2_D_3_, 24R,25(OH)_2_D_3_, and 25(OH)D_3_ into PTEC-DBP media to a final concentration of 0.02, 0.02, 0.2, and 200 ng/mL, respectively. Effluent from the 48-h 25(OH)D_3_ treatment was collected and stored at <−80 °C aboard the ISS U.S. National Laboratory. All treated samples remained frozen throughout the return trip to Earth and shipment to the University of Washington where they were stored at −80 °C until extraction.

Because vitamin D and its metabolites are light-sensitive, all steps were performed under low light. If the treatment sample volume was <500 µL, it was brought up to 500 µL with PTEC-DBP media. Proteins were precipitated by adding 1 mL of 1:1 isopropanol:methanol, vortexing, then incubating at room temperature for 10 min, followed by centrifugation at 16,100×*g* for 10 min. The supernatant was decanted into silanized 16 × 100 mm tubes (Fisher, 12100387) before liquid-liquid extraction by adding 3 mL of 60:40 hexane:methylene chloride. The tubes were capped, shaken on a horizontal shaker for 15 min, and then centrifuged for 10 min at 16,100×*g* in a swinging bucket rotor. The resultant upper solvent layer was transferred to clean silanized glass tubes. The liquid–liquid extraction procedure was repeated twice more, with the resultant upper solvent layer combined into a single tube. After complete evaporation of the solvent under a nitrogen stream at 40 °C the residue was derivatized with 4-(4-(dimethylamino)phenyl)-3H-1,2,4-triazole-3,5(4H)-dione (DAPTAD). DAPTAD stock solution (4 mg DAPTAD in 4 mL ethyl acetate) was diluted 1:1 in acetonitrile, and 200 µL was added to the residue, vortexed, and incubated at room temperature for 45 min with vortex-mixing every 15 min. At the end of the incubation, the samples were dried down under a nitrogen stream at 40 °C, resuspended in 52 µL methanol, and vortexed. 23 µL deionized water was added to the samples before vortexing and centrifuged for 15 min at 16,100×*g* to remove excess DAPTAD and solid precipitate. The supernatant was transferred to amber liquid chromatography vials containing silanized glass inserts. The vials were stored at −80 °C until LC/MS/MS analysis the following day.

### Vitamin D chromatography and mass spectrometry

Chromatographic separation was performed as previously described^47^. Briefly, the method required an RP-Amide (2.1 × 150 mm, 2.7 µm) column (Supelco 2-0943) at room temperature on a Shimadzu Nexera UPLC using water (A, 0.1% formic acid) and methanol (B, 0.1% formic acid) as the mobile phases. Analytes were separated using the following gradient: solvent B starting at 55% for the first minute, increasing linearly to 65% from 1 to 6 min, held at 65% until 8 min, increasing linearly to 75% from 8 to 15 min, held at 75% until 15.5 min, increasing linearly until 90% from 15.5 to 17 min, held at 90% until 23 min, then returning to 55% from 23 to 23.5 min. The injection volumes were 0.3 µL for the analysis of 25(OH)D_3_, while 10 µL was used for all other analytes. Analytes were detected using a positive ionization method on an AB Sciex 6500 QTRAP mass spectrometer (SCIEX, Framingham, MA). The parent and daughter ions were detected using multiple reaction monitoring with channels of *m*/*z* set to detect 25(OH)D_3_ (619.2 → 601.1), 25(OH)D_3_-d_6_ (625.4 → 341.1), 24R,25(OH)_2_D_3_ (635.2 → 341.1), 24R,25(OH)_2_D_3_-d_6_ (641.2 → 341.1), 4β,25(OH)D_3_ (635.2 → 357.1), 1α,25(OH)D_3_ (635.2 → 357.1), 1α,25(OH)D_3_-d_6_(641.2 → 357.1). The retention times for the analytes were as follows: 25(OH)D3, 18.16 min; 25(OH)D_3_-d_6_, 18.13 min; 24R,25(OH)_2_D_3_, 13.4 min; 24R,25(OH)2D_3_-d_6_, 13.34 min; 4β,25(OH)D_3_, 15.06 min; 1α,25(OH)D_3_, 15.3; and 1α,25(OH)D_3_-d_6_, 15.2 min.

### Supplementary information

Supplementary Information

## Data Availability

The datasets generated and/or analyzed during the current study are available in the BioSystics data repository [https://www.biosystics.com/]. In addition, data is publicly available in the NASA Open Science Data Repository at 10.26030/hhwd-v491, OSD-516.
