# Supplementary material for: Modeling cellular responses to serum and vitamin D in microgravity using a human kidney microphysiological system
Source: NPJ Microgravity. 2024 Jul 9;10:75. doi: 10.1038/s41526-024-00415-2 (PMC11233620; doi:10.1038/s41526-024-00415-2)
Supplement: Supplementary file 1 — Supplementary Information [file 41526_2024_415_MOESM1_ESM.pdf]

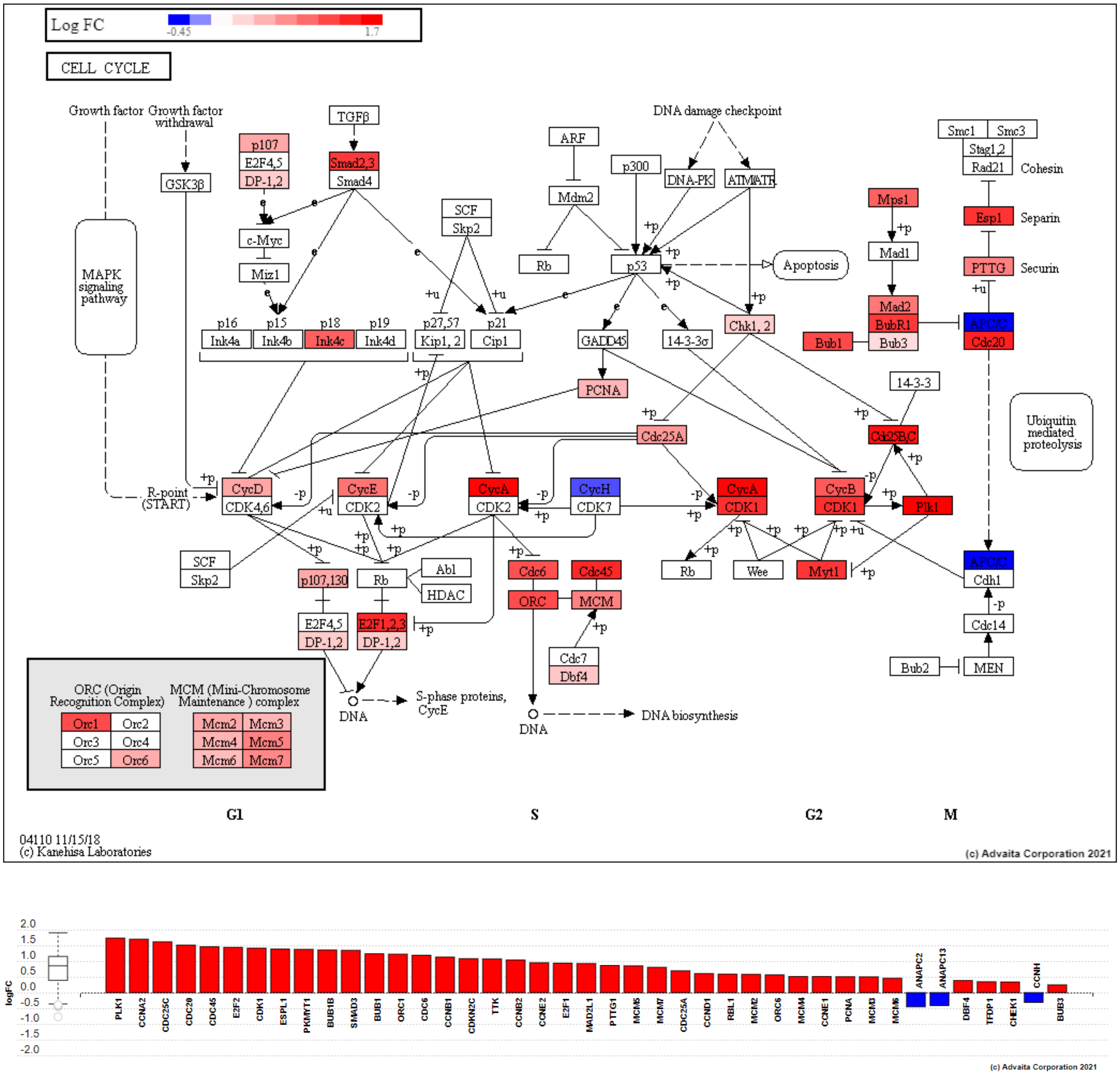

Supplementary Figure 1. Serum induces cell cycle genes in ground condition. Treatment of PT-MPS with 2% normal human serum in the ground condition for 48-hours induces cell cycle genes. Top panel: the position of gene within the cell cycle (i.e., G1, S, G2, M) and their interaction (inhibtion or activation) with other cell cycle associated genes is shown. Bottom panel: The log_2 fold change in gene expression in the serum-treated samples relative to media control samples is displayed.

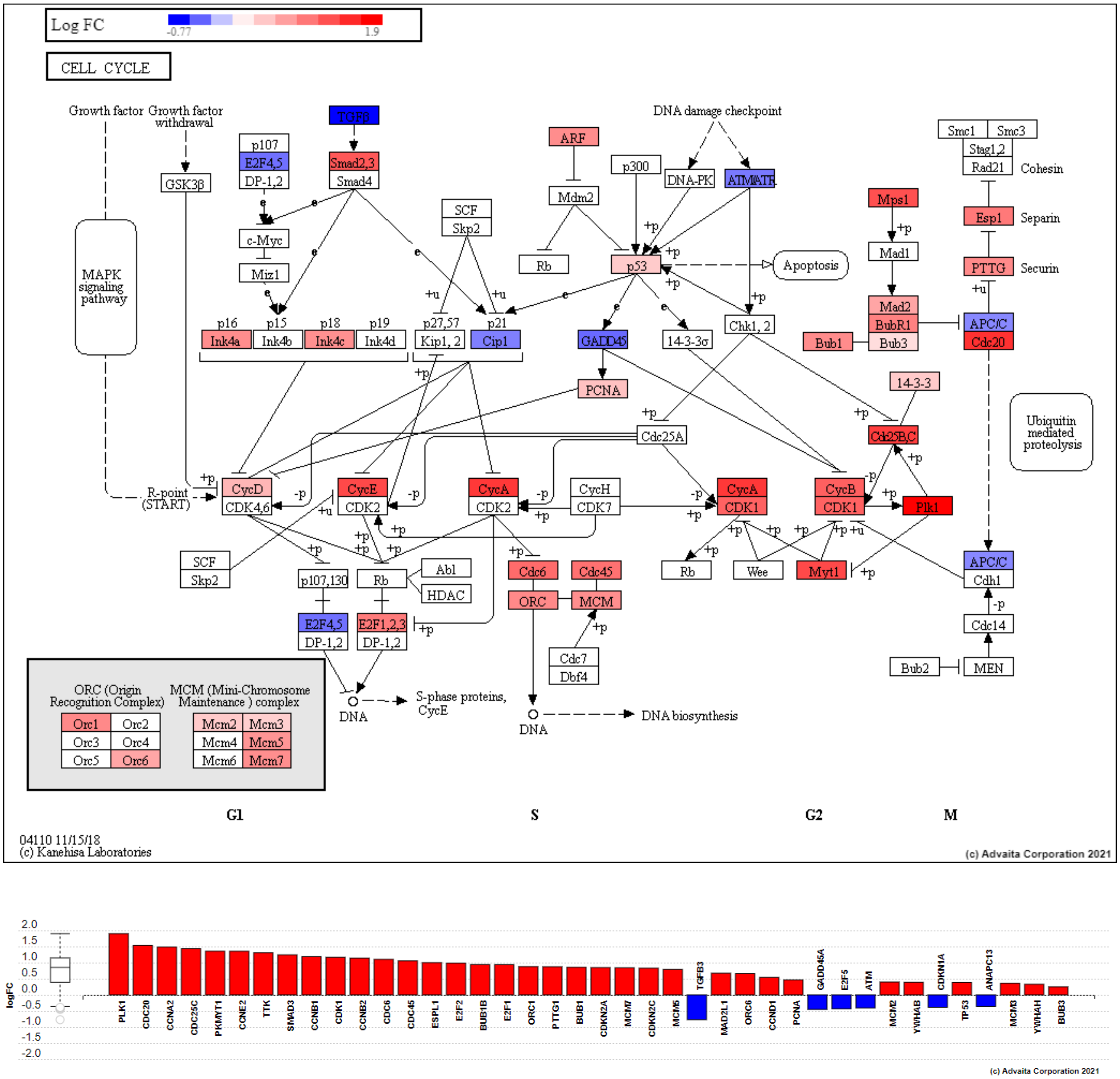

Supplementary Figure 2. Serum induces cell cycle genes in flight condition. Treatment of PT-MPS with 2% normal human serum in the flight condition for 48-hours induces cell cycle genes. Top panel: the position of gene within the cell cycle (i.e., G1, S, G2, M) and their interaction (inhibtion or activation) with other cell cycle associated genes is shown. Bottom panel: The $\log_2$ fold change in gene expression in the serum-treated samples relative to media control samples is displayed.

Supplementary Table 1. Genes differentially expressed in PTECs by 2% human serum treatment in selected pathways in the ground and flight conditions.

		Ground serum vs Ground media	Flight serum vs Flight media
**Pathway**	**Direction**	**Gene**	**Gene**
Cytokine-cytokine receptor interaction	Upregulated	*BMP2, BMPR1A, CCL20, CCL28, CD40, CD70, CSF1, CXCL1, CXCL16, CXCL3, CXCL5, CXCL6, EDA2R, EDAR, FAS, IL18R1, IL1RL1, IL23A, IL32, IL6ST, LIF, LTB, RELT, TGFBR2, TNFRSF11A, TNFRSF11B, TNFSF10*	*CCL2, CCL20, CCL28, CD40, CD70, CLCF1, CSF1, CXCL1, CXCL16, CXCL2, CXCL3, CXCL5, CXCL6, CXCL8, EDAR, IL15RA, IL18R1, IL23A, IL32, INHBB, LIF, LTB, PF4V1, TNF, TNFRSF11A, TNFRSF11B, TNFSF10, TNFSF15*
	Downregulated	*GDF15, IL13RA2, IL17RB, POR, TNFRSF14*	*CTF1, GDF15, GDF7, IL13RA2, IL17RB, IL1RL2, LEPR, LIFR, TGFB3*
Chemokine signaling	Upregulated	*ADCY3, ADCY7, CCL20, CCL28, CXCL1, CXCL16, CXCL3, CXCL5, CXCL6, DOCK2, MAP2K1, NFKB1, PXN, SHC4, VAV1*	*CCL2, CCL20, CCL28, CDC42, CXCL1, CXCL16, CXCL2, CXCL3, CXCL5, CXCL6, CXCL8, DOCK2, HRAS, NFKBIA, PF4V1, PXN, RAC1, SHC4*
	Downregulated	*MAPK3*	*ADCY9, GNAI1, GRK2, MAPK3, SOS1, SOS2*
PPAR signaling	Upregulated	*ACSL5, FABP5, SORBS1*	*ACSL5, FABP5, SORBS1*
	Downregulated	*ACADM, ACSL1, ACSL3, ACSL4, ANGPTL4, CPT1A, DBI, EHHADH, FABP3, FADS2, GK NR1H3, SCD, SCP2, SLC27A2, SLC27A5*	*ACADL, ACADM, ACSL1, ACSL3, ACSL4, ANGPTL4, CPT1A, EHHADH, FABP3, FADS2, GK, ME1, NR1H3, PLIN2, SCD, SCP2, SLC27A2*
Fatty acid metabolism	Upregulated	*ACSL5, HACD4*	*ACSL5*
	Downregulated	*ACAA2, ACACA, ACADM, ACADS, ACADSB, ACADVL, ACAT1, ACAT2, ACSL1, ACSL3, ACSL4, CPT1A, EHHADH, FADS1, FADS2, FASN, HADH, HADHA, HADHB, HSD17B8, MECR, SCD, SCP2*	*ACAA2, ACACA, ACADL, ACADM, ACADS, ACADSB, ACADVL, ACAT1, ACAT2, ACSL1, ACSL3, ACSL4 CPT1A, EHHADH, FADS1, FADS2, FASN, HADH, HADHA, HADHB, HSD17B8, MECR, PPT1 SCD, SCP2*
TCA cycle	Downregulated	*ACO2, CS, IDH2, IDH3G, MDH1, MDH2, OGDHL, PDHA1, SDHB, SDHC, SDHD, SUCLG1, SUCLG2*	*ACO2, CS, DLAT, DLD, FH, IDH1, IDH2, IDH3A, MDH1, MDH2, OGDH, OGDHL, PDHA1, SDHB, SDHD, SUCLA2, SUCLG1, SUCLG2*
Steroid biosynthesis	Upregulated	*CYP24A1*	*CYP24A1*
	Downregulated	*CYP51A1, DHCR7, EBP, FDFT1, HSD17B7, LSS, MSMO1, NSDHL, SC5D, SQLE, TM7SF2*	*CYP51A1, DHCR7, EBP, FDFT1, HSD17B7, LSS, MSMO1, NSDHL, SC5D, SQLE, TM7SF2*

Table displays genes differentially expressed by 48-hour 2% normal human serum treatment relative to control for various pathways in the ground (middle column) and flight conditions (far right column).

Supplementary Table 2. Fatty acid, intermediary, and cholesterol metabolism genes.

		Ground serum vs Ground media		Flight serum vs Flight media	
**Role**	**Gene**	**Log_2FC**	**Adj. p-value**	**Log_2FC**	**Adj. p-value**
Formation of diacylglycerol, lipogenesis	*LPIN1*	-0.40	8.61E-03	-0.49	1.74E-03
FA binding	*FABP3*	-1.55	4.94E-14	-1.38	3.06E-10
FA acyl-CoA ligase activity	*SLC27A3*	-1.17	4.72E-13	-1.21	7.32E-12
Mitochondrial membrane transporters	*SLC25A1*	-0.62	1.02E-09	-0.5	5.43E-06
	SLC25A3	-0.31	7.43E-03	-0.29	3.37E-02
	SLC25A4	-0.65	4.22E-08	-0.73	8.89E-09
	SLC25A5	-0.63	1.17E-09	-0.64	6.33E-09
	SLC25A6	-0.64	2.54E-09	-0.66	8.20E-09
	SLC25A11	-0.45	7.53E-06	-0.46	1.80E-05
	SLC25A16	-0.42	1.02E-03	-0.39	1.39E-02
	SLC25A23	-0.38	2.59E-04	-0.39	5.53E-04
	SLC25A30	-0.71	5.70E-06	-0.98	4.50E-08
Tricarboxylic acid cycle (TCA)	*ACO2*	-0.64	3.87E-07	-0.81	1.17E-09
	CS	-0.34	3.56E-03	-0.48	9.12E-06
	DLAT	-0.27	2.28E-01	-0.60	4.10E-05
	DLD	-0.18	6.49E-01	-0.40	1.73E-02
	IDH2	-0.55	9.06E-06	-0.58	1.14E-05
	IDH3A	-0.27	2.40E-01	-0.42	2.42E-02
	MDH1	-0.45	1.89E-06	-0.52	9.71E-08
	MDH2	-0.37	1.12E-04	-0.39	3.12E-04
	OGDH	-0.23	3.00E-01	-0.54	1.22E-05
	OGDHL	-0.93	1.85E-08	-0.81	6.66E-06
	PDHA1	-0.37	1.12E-04	-0.35	1.14E-03
	SDHB	-0.44	1.04E-05	-0.42	1.33E-04
	SDHC	-0.28	2.11E-02	-0.24	1.46E-01
	SDHD	-0.52	1.61E-06	-0.64	1.00E-06
	SUCLA2	-0.21	4.77E-01	-0.39	1.34E-02
	SUCLG1	-0.49	1.46E-05	-0.59	6.65E-07
	SUCLG2	-0.37	6.92E-03	-0.45	8.14E-04
SREBF2 target genes	*CYP51A1*	-0.86	1.36E-06	-1.00	2.32E-07
	DHCR7	-1.44	3.89E-20	-1.35	4.11E-17
	FDFT1	-0.87	8.11E-16	-0.82	4.89E-13
	FDPS	-0.73	1.97E-09	-0.48	8.03E-04
	HMGCR	-0.43	2.88E-02	-0.68	1.98E-04
	HMGCS1	-0.77	6.14E-03	-1.19	5.94E-05
	IDI1	-1.32	1.42E-14	-1.31	4.89E-13
	LDLR	-1.08	1.43E-11	-1.11	7.25E-11
	LSS	-0.98	6.64E-12	-0.93	5.50E-10

	MVD	-0.74	1.66E-10	-0.55	1.18E-05
	NPC1L1	-0.94	2.31E-04	-1.20	5.52E-05
	PCSK9	-1.25	1.03E-10	-1.46	2.27E-11
	SC5D	-0.65	1.54E-05	-0.73	6.81E-06
	SQLE	-0.82	2.59E-11	-0.73	2.10E-08
	TM7SF2	-0.66	5.23E-09	-0.49	1.34E-04

Genes differentially expressed in PT-MPS after 48-hour 2% normal human serum treatment relative to media control in the flight and ground condition. Numbers highlighted in red are not statistically significant at an adjusted p-value of 0.05. Adj. p-value, adjusted p-value, FA, fatty acid; Log_2FC, Log_2 fold change.

Supplementary Table 3. Top 20 genes up- and down-regulated by serum on ground

Ground serum vs Ground media					
Upregulated			Downregulated		
**Gene**	**Log_2FC**	**Adj. p-value**	**Gene**	**Log_2FC**	**Adj. p-value**
SMAD3	1.35	1.50E-21	*DHCR7*	-1.44	3.89E-20
NCEH1	1.21	4.80E-16	*ACAT2*	-1.24	9.99E-17
FAM149A	1.91	1.83E-13	*FDFT1*	-0.87	8.11E-16
IRF1	0.81	2.29E-13	*MVK*	-1.17	5.61E-15
DHRS3	3.52	1.77E-12	*IDI1*	-1.32	1.42E-14
TGFBI	1.91	2.67E-12	*MMAB*	-0.89	1.88E-14
HMGA1	1.07	3.43E-12	*FABP3*	-1.55	4.94E-14
VSIR	1.97	4.52E-12	*GCAT*	-1.00	2.49E-13
DEPP1	1.08	1.62E-11	*ACADVL*	-0.60	3.47E-13
LINC00511	0.94	1.76E-11	*SCD*	-1.90	3.80E-13
ELK3	0.98	2.74E-11	*SLC27A3*	-1.17	4.72E-13
LAMC2	1.25	2.75E-11	*NSUN5P1*	-1.01	4.91E-13
APOBEC3B	1.48	3.01E-11	*SLC16A7*	-1.54	2.81E-12
LOXL2	1.19	8.55E-11	*HPN*	-1.19	3.12E-12
EPHB2	0.78	1.43E-10	*ACSS2*	-0.87	3.38E-12
PLAUR	1.18	1.49E-10	*EBP*	-1.03	3.82E-12
TPX2	1.31	1.49E-10	*RDH11*	-0.61	3.92E-12
RACGAP1	0.95	4.21E-10	*LSS*	-0.98	6.64E-12
GPRC5A	1.11	4.21E-10	*LDLR*	-1.08	1.43E-11
TFPI2	1.77	5.37E-10	*HAGHL*	-1.00	1.89E-11

Top-20 genes most significantly differentially expressed after 48-hour 2% normal human serum treatment relative to media control in PT-MPS in the ground condition. Abbreviations: Log_2FC, Log_2 fold change; Adj p.value, adjusted p.value.

Supplementary Table 4 Top 20 genes up- and down-regulated by serum in flight

Flight serum vs Flight media					
Upregulated			Downregulated		
**Gene**	**Log_2FC**	**Adj. p-value**	**Gene**	**Log_2FC**	**Adj. p-value**
IRF1	1.25	8.80E-20	*DHCR7*	-1.35	4.11E-17
SMAD3	1.24	1.02E-17	*SCD*	-2.16	9.62E-14
TAGLN2	0.85	3.79E-13	*ACAT2*	-1.15	3.07E-13
PLAU	1.01	1.08E-12	*IDI1*	-1.31	4.89E-13
TNFSF10	1.39	1.95E-12	*FDFT1*	-0.82	4.89E-13
SAT1	0.69	3.19E-12	*SLC16A7*	-1.75	9.83E-13
ELF3	0.74	2.27E-11	*ACSS2*	-0.97	9.83E-13
LINC00511	1.07	2.27E-11	*ACSL1*	-1.71	1.11E-12
APOBEC3B	1.78	3.52E-11	*PLA2G4F*	-1.50	2.72E-12
EPHB2	0.88	3.80E-11	*SLC27A3*	-1.21	7.32E-12
HMGA1	1.07	3.80E-11	*PAIP2B*	-1.38	1.06E-11
DEPP1	1.20	1.15E-10	*PCSK9*	-1.46	2.27E-11
CPM	0.87	1.46E-10	*PPARGC1A*	-1.14	2.27E-11
LGALS9	2.05	1.50E-10	*PANK1*	-1.14	2.66E-11
KPNA2	0.72	1.83E-10	*MSMO1*	-1.26	2.78E-11
TGFBI	1.80	3.06E-10	*UQCRC2*	-0.78	3.87E-11
DHRS3	3.63	3.12E-10	*LDLR*	-1.11	7.25E-11
LCN2	2.09	3.52E-10	*OSBPL1A*	-0.89	9.61E-11
TBXAS1	0.97	5.50E-10	*ATP1B1*	-0.78	9.75E-11
PLAUR	1.23	5.63E-10	*RAP1GAP*	-1.20	1.12E-10

Top-20 genes most significantly differentially expressed after 48-hour 2% normal human serum treatment relative to media control in PT-MPS in the flight condition. Abbreviations: Log_2FC, Log_2 fold change; Adj p.value, adjusted p.value.
